# Towards a Three-Component Model of Fan Loyalty: A Case Study of Chinese Youth

**DOI:** 10.1371/journal.pone.0124312

**Published:** 2015-04-17

**Authors:** Xiao-xiao Zhang, Li Liu, Xian Zhao, Jian Zheng, Meng Yang, Ji-qi Zhang

**Affiliations:** 1 Beijing Key Lab of Applied Experimental Psychology, School of Psychology, Beijing Normal University, Beijing, People's Republic of China; 2 Department of Psychology, University of Kansas, Lawrence, Kansas, United States of America; Center for BrainHealth, University of Texas at Dallas, UNITED STATES

## Abstract

The term “fan loyalty” refers to the loyalty felt and expressed by a fan towards the object of his/her fanaticism in both everyday and academic discourses. However, much of the literature on fan loyalty has paid little attention to the topic from the perspective of youth pop culture. The present study explored the meaning of fan loyalty in the context of China. Data were collected by the method of in-depth interviews with 16 young Chinese people aged between 19 and 25 years who currently or once were pop fans. The results indicated that fan loyalty entails three components: involvement, satisfaction, and affiliation. These three components regulate the process of fan loyalty development, which can be divided into four stages: inception, upgrade, zenith, and decline. This model provides a conceptual explanation of why and how young Chinese fans are loyal to their favorite stars. The implications of the findings are discussed.

## Introduction

“Pop culture is ubiquitous in contemporary society.” [1] Pop fans, the persons who are enthusiastically devoted to pop stars, are regarded as the consumers of the pop industry [2] or an important sphere for the consumption of pop culture all around the world. For example, Michael Jackson, the King of Pop, died on June 25, 2009. Up to one million of his fans attended his funeral ceremony [3]. During his world tour from 1996 to 1997, Jackson performed 82 concerts in 58 cities to over 4.5 million fans [4]. TVXQ, a South Korean pop duo, have a group of fans called “Cassiopeia.” According to news sources in 2008, the “Cassiopeia” was listed in the Guinness Book of Records as “the world’s largest fan club” with more than 800,000 members [5]. Obviously, pop fans, as an indispensable force for pop culture, cannot be ignored by researchers.

In the wake of globalization and the rapid development of mass media, youth culture in China has joined the global trend of becoming commercialized, which is manifested in the rise of popular culture [6–7]. As a consequence of this transition, the fan population of pop stars has exploded over the last decade. While the actual figure of the fan population in China is uncertain, the case of the fans of Chris Lee, a Chinese pop singer, illustrates the popularity of fandom among Chinese youth. Chris Lee received over 10 million votes from her fans in the final round of the Super Girl's Voice contest of China. She was the first superstar chosen by fans, and she was rewarded the honorary title of “Asia Hero” by Time magazine in 2005. Her fans are known by the collective name “CORN.” The “CORN” set up a charitable fund and collected over one million dollars to help children with leukemia [8], which is a creation of public welfare in China. We are not only astounded at the power of the fans but are also curious about why and how fans remain loyal to their favorite stars.

The term “fan” in everyday language describes a range of interest from the casual follower to the obsessed person [9]. Researchers try to distinguish fans from followers [10] or from consumers [11]. The critical characteristics of “fans” are that they are not just passive recipients of the media product, but are active participants within fandom as a social, cultural and interpretive institution [9–11]. Researchers also identify a number of motivations for becoming a fan: romantic attraction, physical attraction, identification with celebrity, hero worship and task attraction etc. [12–13]. In the field of fan psychology, various studies have explored specific psychological features of celebrity worshippers [14–18]. The term celebrity worship is originally coined as an abnormal type of parasocial relationship that is driven by absorption and addictive elements [19]. However, recent research indicates that it is best perceived as a continuum phenomenon, ranging from normal admiration to the psychopathological [18].

The fan phenomenon has been increasingly approached from the perspective of loyalty [20–22]. The term “fan loyalty” thus gains its legitimacy to depict the loyalty felt and expressed by a fan towards the object of his/her fanaticism in both everyday and academic discourses [23]. However, there are endless debates over the concept of loyalty [24–25]. It is generally accepted in the field that there is no commonly agreed upon view of loyalty. Most researchers investigate this topic in the context of consumption [26–29]. Some researchers focus on separating the behaviors and minds of people when alluding to the topic of loyalty [30]. Some researchers consider simple behavioral standards, such as repeat purchases [31], to illustrate the degree of loyalty. We suggest that the meaning of loyalty varies with individual circumstances and should be discussed for a specific area. Concerning the fan phenomenon, most studies focus on the domain of sports [26, 32–33]. To be clear about the pop fan phenomenon, the meaning of fan loyalty is thus worthy of further exploration from the background of pop culture. To understand the meaning of fan loyalty, we must return to its basics: the components of fan loyalty and the process of loyalty development that is regulated by these components.

According to social exchange theory, loyalty is regarded as a process [33–34]. Hence, pop fans’ experiences are important to the exploration of the concept of fan loyalty. Following social exchange theory [35–37], some researchers have paid close attention to the exchange relationships that accrue during the process of fan loyalty development [9, 27, 38–39]. However, previous research has focused only on those who were loyal and neglected those who had been loyal but were no longer loyal at the time of sampling [21]. The termination of fan loyalty is excluded in most research. The entire chain of fan loyalty development is thus undermined. To fill this gap, we must understand the basic components of fan loyalty.

The overall aim of this study was to explore the meaning of fan loyalty for young people in the context of the fan phenomenon in China. This aim is subdivided, analytically, into the following two research questions: (1) What are the components of fan loyalty? (2) How do these components regulate the process of fan loyalty development?

## Method

### Ethics Statement

The study was reviewed and approved by the Committee of the Protection of Subjects at Beijing Normal University. All participants provided written informed consent before the study, and they were fully debriefed at the end of the interview according to the established guidelines of the committee.

### Participants

The participants were recruited from a popular website for young people in China (www.oiegg.com). We posted the recruitment information on the website as follows: “*A social psychology team at BNU is conducting a research project on fans behavior*. *We need to recruit some volunteers to participate in face-to-face interviews*. *No matter you “are” or “were” a fan*, *you can contact with us for participating in this study*. *The selected participants will receive 40 yuan as a reward for their participation*.”

In total we received the response from 28 individuals. For the purpose of screening the formal interviewees, we asked these potential participants to answer the following questions: “Do you perceive yourself as a fan?”, “Who is your favorite star?”, “How long have you been a fan of the star?”, “Have you attended in the fan club and participated in its activities?”, and “Have you ever seen the star personally?” The criteria for the screening were as follows: 1) being or once being a fan of a pop star, 2) being or once being a member of a fan club, 3) having participated in some activities organized by a fan club. Finally 16 participants were selected to participate in this study. The details of the participants are shown in Table 1.

**Table 1:** Details about the Fan Participants.

Participant	Age	Gender	Length of Being a Fan (months)	Direct Contact With Star (yes/no)	Still A Fan (yes/no)
01	22	Female	15m	yes	yes
02	21	Female	12m	yes	no
03	23	Female	37m	no	yes
04	22	Female	42m	yes	yes
05	19	Female	12m	no	yes
06	21	Female	90m	yes	no
07	20	Female	29m	no	yes
08	20	Female	63m	no	yes
09	21	Female	7m	no	yes
10	22	Female	15m	yes	yes
11	24	Female	27m	yes	yes
12	24	Male	21m	yes	yes
13	21	Female	104m	yes	yes
14	25	Female	24m	yes	yes
15	23	Female	132m	yes	yes
16	24	Female	40m	yes	yes

As shown on Table 1, the sample was young people aged between 19 and 25 years and comprised a large proportion of females. This coincides with the fact that the community of pop fans is dominated by young women [40–42]. Indeed, the sample size is small in the present study. However, samples for qualitative studies are generally much smaller than those used in quantitative studies. A small sample (less than 20) facilitates a researcher’s close association with participants, and enhances the validity of an in-depth inquiry [43]. To maximize the data saturation [44], a variety of participants including those with or without direct contact with a star, with different lengths of time of being a fan, and those who were still or were no longer a fan at the time of the interview were included in this study.

### Data collection

One week prior to the formal interview, each participant was asked to recall his or her fan experiences and to select approximately ten pictures which could chronicle his or her whole experiences as a fan. These pictures included the logos of their own fan clubs, photos of activities of the fan club members, or photos from a concert of their favorite star. The participants were asked to email the pictures to the researcher prior to the interview.

The interviewer (Xiao-xiao Zhang) was a PhD student who had completed a qualitative research course and had been trained on the in-depth interview method. The interviewer was without any bias or assumptions on the research topic. The interview guide was pilot-tested by the interviewer prior to the formal interviews.

Face-to-face in-depth interviews were used to obtain data for this study. The interviews were conducted in a quiet chat room at Beijing Normal University. There was no other person in the room except for the interviewer and the participant. The interviews were audio-recorded, and the audio recordings were permitted by the participants. The prepared printed pictures were used to assist the interview. The interviewer made field notes during the interview to prevent data loss. During each interview session, the participant was asked to arrange the prepared pictures in chronological order and to depict his or her fan experiences based on the pictures. During the interview, the researcher made detailed inquiries of the participants’ statements made in response to the research questions. The interview questions were organized around the following areas: 1) the process or procedure of becoming a fan; 2) the role and impact of the fan club and the star on fans’ activities; 3) the effort put into fan activities and the rewarding experience of being a fan; 4) attitude changes in the entire process; 5) the reasons for the continuation or termination of fan activities (see S1 Appendix: Interview guide). The participants’ personal information, such as age and the length of time of being a fan were also collected at the end of the interview. Each interview lasted approximately one to one and a half hours. The verbatim transcripts of the audio-recorded interviews were returned to the participants after the interview to make sure there was not any misunderstanding during the interviews. None of the participants had an objection to the transcripts. Hence, the data were reliable for analysis.

### Data analysis

Recall that the purpose of this study was to explore the meaning of fan loyalty in young people. To achieve this aim, grounded theory was selected as an appropriate approach for data analysis in this study. This choice was based the consideration that grounded theory differs from other methods of qualitative data analysis in that its end goal is to develop theory inductively from data, rather than to test existing theories or generate only themes [45–47]. Following the procedures suggested by grounded theory [46], open coding, axial coding, and selective coding were adopted to analyze the transcripts of the audio-recorded interviews, and to develop a conceptual model of fan loyalty. During the stage of open coding the main categories of the transcripts of all interviews were identified and labeled. For example, the conceptual categories “devotion for fan club” and “pursue higher position” were emerged from our raw data at this stage. During the stage of axial coding the conceptual categories were related to each other, via a combination of inductive and deductive thinking. For example, we found the purpose of “devotion for fan club” was to “pursue higher position”, a causal relationship between the two categories was thus established at this stage. During the stage of selective coding one category was defined as the core category and all other categories were related to the core category. For example, the category “loyalty” was chose as the core category around which the other categories were grouped for the purpose of explaining the phenomena of fan loyalty and its development at this final stage.

In the process of the data analysis, all transcripts were read word by word and were re-read several times by the researcher to obtain a more appropriate label. For each data analysis step, the labeling process was revisited for validation and to incorporate newly uncovered concepts. The pictures collected from the participants were also used as aid materials to assist with the transcript analysis. The software package NVivo 8 was used to assist the transcript analysis. The coding scheme was continually created and revised throughout the coding process with an aim towards balancing all contents and constructs. The interviewer coded the whole data. Another coder independently coded the half of the randomly selected transcripts. The inter-coder agreement [48–49] is 0.82. The two coders discussed and resolved differences when there were inconsistencies in coding between them.

## Results and Analysis

### The components of fan loyalty

Fan loyalty was not explicitly expressed by the participants as a certain construction but was used as a short hand for heterogeneous meanings. Discourse about their fan experiences invoked a wide range of themes, which varied from one individual to another. Despite the great complexity evident in the data, data analysis revealed that fan loyalty is a triadic construction structured around three pivotal components: involvement, satisfaction, and affiliation.

#### Involvement

In the context of fan loyalty, involvement refers to the behavior and emotion devoted by a fan to the recipient of his/her fanaticism. It involves a set of activities performed by the fan individually and in a group setting.

A fan voluntarily collects all types of information on the star. Indeed, the information acquisition is a type of reinforcement for the involvement of the fan. On the one hand, it stimulates a fan to consume the products of the star, and on the other hand, it intensifies a fan’s adoration of the star to which they are faithful.

*Excerpt 01*

Participant 01: I have learned about him since watching a video on the Internet. The more I knew about him, the more I loved him dearly. The more videos I watched, the more I would like him.

*Excerpt 02*

Participant 06: I checked lots of information about him by myself, and I felt that I liked him very much. […] I have heard all his songs, and watched all of his videos.

Obtaining information on the star appeared to be much more important for fans in terms of their involvement. A fan learned about the star using many different channels. Fans spent considerable time searching for information on the star. The constantly updated information on a star provides an opportunity to obtain more fans. Furthermore, in many circumstances, there were some competitions and comparisons between stars. To maintain and even to promote the status of his/her favorite star, a fan would make much effort to support the star, such as boosting and voting for the star.

*Excerpt 03*

Participant 12: There were two vocal concerts last week. One was performed by our favorite Stefanie Sun, the other was performed by Wang Lee Hon, who is also an excellent singer. Our concert was a packed-house event. We bought almost all of the tickets! We were so happy that night! However, this was not the case for the other concert.

The success of a star relies on the shared responsibilities between fans and their star. From the viewpoint of fans, due to the “basking-in reflected-glory”[50], they recognize the responsibilities towards their favorite star. They hope their star is better than others. Hence, they always exert great effort to consolidate their star’s position, particularly compared with other stars. The meaning of loyalty to a star is comprised not only of consumption but also of supporting all things concerning the star. These actions cost time and money and have even resulted in some fans losing their jobs.

The best way for a fan to obtain more information on their favorite star was to find a “group” such as a fan club. Joining a fan club and interacting with other members of the fan club was a typical activity.

*Excerpt 04*

Participant 12: He (another fan in the club) spent all day at our fan club. “Bird’s net” (the name of this fan club) has become a very important part of his life. As far as I know, he has been focusing on it all the time, apart from his job. It is very likely that lots of fans’ normal work is shelved.

There were two major forms of interaction among the members of a fan club: virtual communication and face-to-face contact. The former involved in the exchange of ideas about their favorite star between the member via a web chat room or web forum; the latter was involved in the participation in activities organized by the fan club, such as the celebration of the star’s birthday.

### Satisfaction

In the context of fan loyalty, satisfaction refers to the positive, as opposed to negative, affect that fans experience during the process of interacting with the star and other members of a fan club. It is determined by the fan’s comparison of his/her actual rewards with the costs to the star and the fan club.

A fan always predicted the possibilities of contacting the star directly. As Excerpt 05 shows, the fan compared her rewarding experience of meeting the star to the travel costs in time and money.

*Excerpt 05*

Participant 04: At that time, I never expected to meet him in my life, but it’s really amazing this time in Beijing. […] It’s wonderful I was able to see him finally. He was likely to go to the VIP channel, but I still thought I had a chance to meet him that time. Then, I traveled to the airport from far away. I felt worthy of doing that, I saw him finally! I will come to meet him if there are other chances.

Such a comparison would also occur between a fan’s input to a fan club and the fan club’s return. The fans expected that the fan club could provide them with extra chances to meet the star as a result of their contributions to the club.

*Excerpt 06*

Participant 02: The fan club promised to let us meet the star closely on the condition that we help to set up the location early in the morning. Given this, we made the greatest effort to do the work, but in the end, we didn’t get the concert tickets! At that time, one of my acquaintances from the fan club and I decided to quit, and we will not attend such activities any more.

It is important for a fan to obtain responses from the star. A response, such as a call from the star, was akin to a boost. It made fans feel that doing anything for the star was worthwhile. Fans were also proud of the responses from the star. The responses from the star helped fans make the assessment that their devotion was not in vain. Responses from the star can satisfy fans.

*Excerpt 07*

Participant 11: On my birthday, she (the star she liked) called me on the phone. I thought it’s enough because this would not happen to other fans. If a feedback from the star appeared while I hesitated, I would continue.

The fans regarded a star’s achievement, such as winning a prize, as a type of feedback for them. The star’s achievement confirmed the fan’s choice in being a fan. The fans could thus have a sense of accomplishment. Hence, the star could not only gave a fan some work (e.g., music, a movie) but also self-affirmation.

*Excerpt 08*

Participant 03: She won the Best New Artist at the Hong Kong Film Awards on the 13^th^ of April last. I think it’s really a great achievement. She shot the movie and won the prize. It’s my accomplishment. It pleased me. My efforts were not in vain. She is a rising star, which also enhances my loyalty to her. She lives up to our expectations.

Obtaining the expected feedback from the star or the fan club could satisfy a fan. They could also strengthen a fan’s willingness for further devotion to the star or the fan club.

### Affiliation

In the context of fan loyalty, affiliation indicates the relationships between fans and the fan club they joined. Like the importance of community to consumers [51–53], the fan club is particularly important to a fan. A fan club has an incredible hierarchy and is strictly regulated. Junior fans that have just joined a fan club obtain little related information and have little influence on the star or the fan club. However, senior fans or “big fans,” the core members of a fan club, obtain “first-hand” information, have great influence on the group, and also devote much to the group and the star. Typically, senior fans have much more experiences of contact with the star and are at higher levels of fan loyalty.

*Excerpt 09*

Participant 13: Different fans had different positions in the “hierarchical chain.” Some could go to Japan. Someone would be very haunted because he or she did not have enough money. You must get along well with those who can go Japan. There’s a hierarchy system, and also “opinion leaders”…I thought every group is the same. There are some big fans that are familiar with the agent of the star. They can have email discussions on related issues regarding a vocal concert in China. I think I’m at a level just lower than the big fans. There are also many fans at lower levels than me. It may relate to the city you live in, financial situation, information channels, and so on.

*Excerpt 10*

Participant 14: The “big fans” can do more difficult things than us. We as junior fans could only go to the airport to pick the star up, so that we could meet with the star. However, they can get in close contact with the star, and can also contact the agent of the star… You feel they are really very superb.

A fan club was a type of a “hierarchical chain.” The fans at different levels in a fan club received differing degrees of benefits. Senior fans controlled contact with the star could even go abroad to meet with the star. However, junior fans might only receive second-hand “welfare” from the senior fans. As Excerpt 09 shows, this fan’s position was lower than that of “big fans” and higher than many other members in the fan club. There were different levels in a fan club. Fans’ positions in a fan club were influenced by their age, location, spending power, information channels, expertise, and the relationship with other higher-level fans. This pattern is similar to that observed in a previous study [54] where members of a car club were divided into several levels based on their interests, participation, knowledge, and degree of contact with cars of that brand. However, our study further showed that when a fan wanted to have a higher level in the fan club, he/she tried to have good relationship with the bigger fans. It was necessary for a fan to consolidate his/her positions in the fan club because a fan in a higher position received benefits, such as the chance to meet with the star.

*Excerpt 11*

Participant 11: When I run this web forum of fans, I know I should make an effort to bring all members together, even though I was wronged by other members in the fan club. Although I enjoyed the desire of power, I spent much time and energy in preserving the position. It’s a long story.

*Excerpt 12*

Participant 10: I was a junior fan at that time. When we were in the audience at a show of our favorite star, I could only sit in the back. However, the big fans could sit in front of us, and even contacted the star on the stage. I also wanted to go to the stage and have close contact with the star, but what else could I do? I could only obey their arrangement and try to become a big fan.

A big fan enjoyed his/her status, and he/she also needed to devote time to the club to consolidate this position. When a fan wanted to consolidate his/her position in a fan club, he/she contributed time and energy to the club. A fan, particularly a “big fan,” had obligations in the fan club. In turn, the obligations regulated a fan’s behaviors. A junior fan had few privileges, which made him/her desire to upgrade their status. A higher status in the fan club was important for the fan to obtain some privileges, such as contact with the star. In a word, whatever entrenching or enhancing the status in the fan club, they were all able to influence the affiliation of a fan with the fan club.

*Excerpt 13*

Participant 09: I was envious of those who were able to attend the activity!

*Excerpt 14*

Participant 16: Six members paid a lot of money to get to the VIP area, and the others, such as me, could only get non-VIP area tickets. On the way back, unexpectedly, those six members didn’t want to sit near us on the train! One of them even shouted, “how can I take a seat next to these non-VIP-area fans?” It seemed that we were crowded out of the group, and I felt so bad.

Different social classes led to social comparisons [55]. Because there were different levels in a fan club, the comparisons between the fans from different levels were inevitable. The social comparisons between the fans in a fan club typically led to two results: envy of others or being frozen out. The negative outcome between fans was a type of double-edged sword to the affiliation. On one hand, this negative relationship might induce a fan to obtain good status in the fan club. On the other hand, it could undermine a fan’s enthusiasm and remove his/her affiliation with the fan club.

### The process of fan loyalty development

**Fig 1:**
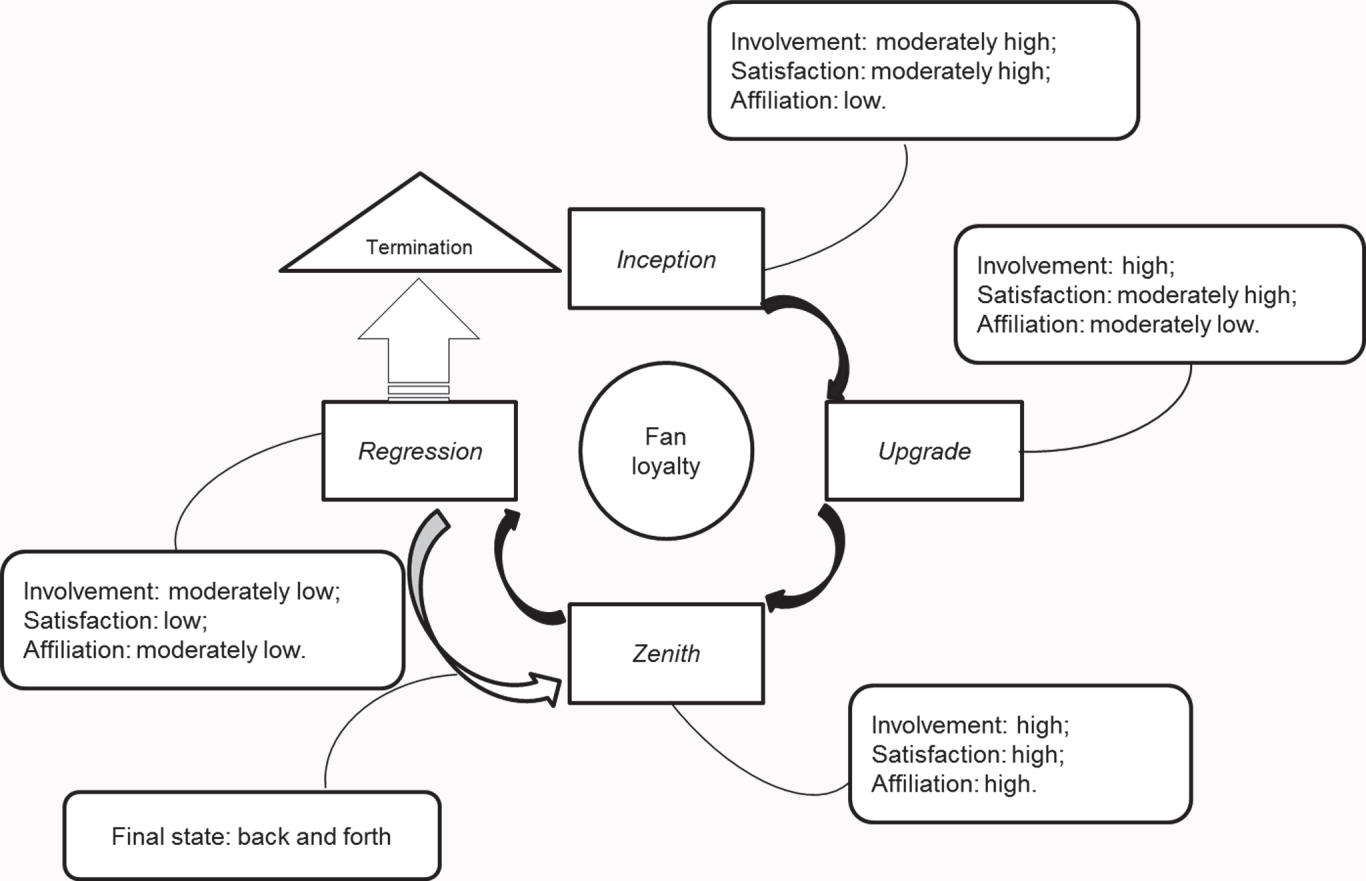
An Interpretative Framework of Fan Loyalty Development.

We investigated the concept of fan loyalty by participants’ expression of their fan experience. According to the data, during the process of fan loyalty, the degree of the three components varied continuously. Changes in these components led to the development of loyalty. Data analysis further revealed that the three components comprising involvement, satisfaction, and affiliation regulated the process of fan loyalty development, which can be divided into four stages: inception, upgrade, zenith, and decline, as shown in Fig 1.

### Inception

When an individual becomes a fan of a pop star, the inception stage begins. During this stage, the fans are typically keen to learn information from the media and have just joined a fan club, and they were referred to as “rookies.” Using this channel, these new fans received much more information on the star from other members of the club, which, in turn, reinforced their fan loyalty process. At this stage, fans became more and more involved in activities relating to the star that they adored. They were moderately highly satisfied with their identity as fans although they were junior members and had a low affiliation with the fan club.

*Excerpt 15*

Participant 14: I was only a rookie when I attended the web forum. The information I got was all from those big fans. However, we were happy to be in a fan club and spent a great deal of time on the web forum every day.

### Upgrade

When moving from the inception stage to the upgrade stage, fans were active in the fan club. At this stage, the fans received more benefits, such as more opportunities to interact with other fans in the club. Because they were not at the highest level in the fan club and had moderately low affiliation, they wanted higher status in the fan club. Although they already had some opportunities to get in touch with the star, they showed only moderately high satisfaction. They realized that the bigger fans would have more privileges and would not hesitate to pay the cost of becoming a big fan. Therefore, during this stage, the degree of their involvement was high.

*Excerpt 16*

Participant 08: I was popular at that time. The small fans supported me. The big fans drew me to their side because they had been divided into two factions. I could do everything that a big fan could, except contacting the broker. I really mixed well in the club. I dealt with the photos for the club and could obtain some privileges.

### Zenith

After the upgrade, fans had almost reached the highest status that they could achieve in the fan club with high affiliation and could obtain lots of benefits from the star and the fan club. Thus, during this stage, a fan could get what he/she wanted as a fan and had reached the zenith of his/her loyalty process. During this time, a fan experienced much involvement and was highly satisfied with the feedback to his/her devotion or his/her situation.

*Excerpt 17*

Participant 15: I had achieved all of the things that could be achieved by fans. When you were a small fan, what could you do? Just looking at him, and interacting with him at most at a distance, which was just a piece of cake for me.

### Decline

Few fans persisted in the zenith over a long period of time, as shown by Excerpt 18. After zenith, fans questioned whether the benefits satisfied him/her. As shown by the data, the relationship between a fan and the star remained as “a distant friend” at best, and the star could never be a real friend of the fan. Additional devotion could become a wasteful cost. When a fan came to this point, the level of satisfaction declined in parallel with moderately low involvement and affiliation. There are typically two types of endings to this stage: termination of loyalty or “back and forth.” The termination of loyalty indicated that the fans had no expectations of further affiliation or further benefits; hence, they discontinued their loyalty. “Back and forth” indicated a final state of an ideal loyal fan: although there was a drop in the degree of the three components since zenith, there was a bond between the fan and the star or the fan club. This bond might prevent a loyal fan from terminating loyalty. For a loyal fan, the decline was similar to a type of rest, like “good rest can work well.” After the decline, a loyal fan would recover the three components to some degree. However, due to the qualifications that a loyal fan already had, he/she could rebuild the affiliation well and quickly. Thus, if the decline to zenith was regarded as a type of continuous process in the degree of involvement and satisfaction, the final state of an ideal loyal fan might be “back and forth” from the decline to the zenith.

*Excerpt 18*

Participant 02: No one can maintain loyalty that long for a star. If there was a kind of final state, it must be a kind of “back and forth.”

*Excerpt 19*

Participant 06: He is just like a distant friend. The relationship between the star and you won’t be better than you expected and he won’t be on call for you.

## Discussion

Loyalty is an elusive concept. As reviewed in the Introduction section, many researchers have paid great attention to the topic over recent decades, but there has been no generally accepted view of loyalty. Indeed, it appears that there is much to be identified on the much lauded but little understood concept of loyalty [56]. Recently, the fan phenomenon in the pop industry has broken into the public view in China [57] and beyond [58]. This study provides a new entry point for the exploration of the fan phenomenon and a discussion of the meaning of loyalty.

This study extends previous research in the examination of components of fan loyalty and its development process. Based on qualitative interviews with young pop fans, we developed a three-component model of fan loyalty. The main tenets of the model are that it elaborates both on the nature of fan loyalty and the development of fan loyalty. Fan loyalty embraces three components: involvement, satisfaction, and affiliation in the model. These three components regulate the process of fan loyalty development, which can be divided into four stages: inception, upgrade, zenith, and decline.

It is valuable to clarify the meanings of the three components of fan loyalty in relation to previous studies. The involvement component in our model refers to the behavior and emotion devoted by a fan to the star of his/her fanaticism. It is highly relevant to the attitudinal and behavioral dimensions of fan loyalty in sports [59]. Behavioral involvement and emotional involvement typically appeared together. It is thus unnecessary to distinguish between the two, and they can be combined for the involvement component. The factors that influence involvement have typically been classified into three categories [60–61]: personal factors (e.g., the same product had different levels of involvement among individuals) [62], physical factors (e.g., the same message delivered via different media influenced consumer responses) [63], and situational factors (e.g., the purchase situation influenced the involvement) [64]. The findings in our study support these three categories, which are embodied by the relationship between the involvement component and the affiliation component. Specifically, fans with different degrees of affiliation typically have different degrees of involvement. This categorization involves the personal category (different levels of involvement among individuals), physical category (different levels of fans in the club had different privileges or information that could influence fan involvement), and situational category (the fan club was an important situational factor that influenced fan involvement).

The satisfaction component in our model refers to positive versus negative effects that fans experienced in the process of interacting with the star and other members of a fan club. It is somewhat similar to the perception of the pleasurable fulfillment of a service [56, 65]. A star in the pop industry does not provide a simple service because the star is not goods in a window. As a person, the star can interact with his/her consumers or fans. A fan club also plays an important role in the process of celebrity worship. The satisfaction component of fan loyalty is thus based on a fan’s comparison of his/her actual rewards from the star and the fan club and the costs to the fan. Several studies have explored the relationship between customer satisfaction and repeat purchases [66–67]. Our findings confirm that fan satisfaction can influence his/her further involvement, which provides us with some evidence of the relationship between these two components of fan loyalty.

The affiliation component in our model refers to the relationship between fans and the fan club they joined. There is a need to explore fan loyalty from both the individual and community perspective [56]. More importantly, the affiliation component not only focuses on the role of the fan club but also illustrates the hierarchical nature of the fan club. A fan club is actually a type of mini-society with different “classes,” which leads to social competition between the fans and involves playing up to the leader character, pursuing rights, and undertaking obligations.

The three components of fan loyalty also differ in their contribution across the lifespan of a fan. During the inception stage, there is moderately high involvement, moderately high satisfaction, and low affiliation. By the upgrade stage, there is high involvement, moderately high satisfaction, and moderately low affiliation. By the zenith stage, there is high involvement, high satisfaction, and high affiliation. By the decline stage, there is moderately low involvement, low satisfaction, and moderately low affiliation. Following Duck’s [68] dissolution process model of the buyer-seller relationship, we found that the termination of fan loyalty derives from low satisfaction of a fan’s costs, which outweigh his/her benefits. Social exchange theory [35–37] suggests that the benefits must be a primary aim for a fan during the development of loyalty. However, past studies have indicated a number of benefits for fans [69]. Our study has further examined the change in benefits required by a fan during different stages of his/her loyalty. Specifically, during the inception stage, a fan requires first-hand information on the star and the identity as a fan. At the upgrade stage, the benefits a fan desires extend to meeting with the star in real life, and the fan attempts to obtain a higher position within the fan club. By the zenith stage, the benefits that a fan may pursue include a closer relationship with the star, like a friend. By the decline stage, a fan perceives no further benefits from the star or the club, and he/she may exit from the loyalty process. In conclusion, fans have different needs at different stages and the growth of a fan requires different types of fulfillment.

Another advantage of this study is that it focuses on the network organized by the fan, the star, and the fan club in discourse by the participants. The construction of the meaning of loyalty in the participant’s minds derives from this triangular perspective. In the field of fan psychology, there are other conceptual models in understanding fan behaviors. For example, the theory of celebrity identification focuses on the psychological attachment from fans to star [70]; the studies of parasocial interaction depict the relationship between media users and media figures who are not “really there” [71–72]. The present study expanded upon previous studies that simply focused on a one-way relationship from fans to star. Our findings indicate that the fan club was essential to the fan by not only providing an identity for the fan but also making links between the fan and the star. In parallel, the fan also had a direct connection with the star. During the investigation of the meaning of fan loyalty, this tripartite relationship became obvious.

No research has ever been performed without limitations; the present study is not an exception. Firstly, the current situation of the pop fans in China is mostly seen in female youth, as a result of which females represent a very great proportion in the sample. However, we tried to achieve the data saturation [44] by collecting participants diversely from various criteria (such as different lengths of being a fan, having or not having direct contact with the star, and whether the fans’ loyalty is terminated). Second, the model of the fan loyalty process developed from the present study describes a general picture of the fan phenomenon, and it thus may not apply for every individual fan. Nevertheless, we explore the fan loyalty process, which aims to help understand the fan phenomenon and learn about the general pattern of fans’ experiences. The typicality of the sample is reached at the expense of the quantity of the sample. Moreover, the applicability is always a salient “characteristic” of the qualitative study. More importantly, the present study provides a baseline for developing a more structured interview schedule and even a questionnaire to enhance the understanding of fan loyalty or consumer loyalty. These tasks remain for the agenda of future studies.

## Supporting Information

S1 Appendix Interview guide. (DOCX)
