# Supplementary material for: Towards a Three-Component Model of Fan Loyalty: A Case Study of Chinese Youth
Source: PLoS One. 2015 Apr 17;10(4):e0124312. doi: 10.1371/journal.pone.0124312 (PMC4401450; doi:10.1371/journal.pone.0124312)
Supplement: S1 Appendix — (DOCX) [file pone.0124312.s001.docx]

**Interview guide**

1. General experiences as a fan

1.1. Please describe when and why did you regard yourself as a fan?

1.2. Please describe how did you catch up on information of your favorite star, and connect to the star?

1.3. Please describe what did you mainly do as a fan? What did you think of these behaviors of you?

2. The role of a fan club in fan loyalty

2.1. Please describe when, how, and why you joined in your fan club?

2.2. What kinds of fan club activities did you usually perform, and why? Please elaborate on them.

2.3. What did you think of fan club and its activities? What are the roles of the fan club in your whole experience as a fan? Please elaborate on them.

3. Devotion and reward of a fan

3.1. Was there anything a fan had to do for the star or the fan club? Please elaborate on them.

3.2. Was there anything a fan could obtain from the star or the fan club? Please elaborate on them.

3.3 Please describe what you think of a fan’s devotion and reward.

4. Attitude changes during the process of fan loyalty

4.1. Please describe the changes of your attitude toward the star or the fan club in your whole experience as a fan.

4.2. Please describe why you changed your attitude toward the fan or the fan club?

5. Termination of fan loyalty

5.1. Are you still a fan?

5.2. (If No) Please describe why you were no longer a fan?

5.3. (If Yes) Have you thought you would no longer be a fan, and why?

6. Additional questions

6.1. Is there anything else you would like to add?

6.2. Do you have any experiences that cannot be reflected by the pictures? What are they?
